# The Endosymbiont *Wolbachia pipientis* Induces the Expression of Host Antioxidant Proteins in an *Aedes albopictus* Cell Line

**DOI:** 10.1371/journal.pone.0002083

**Published:** 2008-05-07

**Authors:** Lesley J. Brennan, B. Andrew Keddie, Henk R. Braig, Harriet L. Harris

**Affiliations:** 1 Department of Biological Sciences, University of Alberta, Edmonton, Canada; 2 School of Biological Sciences, University of Wales, Bangor, United Kingdom; 3 Department of Biology and Environmental Science, Concordia University College of Alberta, Edmonton, Canada; Duke University, United States of America

## Abstract

*Wolbachia* are obligate intracellular bacteria which commonly infect arthropods. They are maternally inherited and capable of altering host development, sex determination, and reproduction. Reproductive manipulations include feminization, male-killing, parthenogenesis, and cytoplasmic incompatibility. The mechanism by which *Wolbachia* avoid destruction by the host immune response is unknown. Generation of antimicrobial peptides (AMPs) and reactive oxygen species (ROS) by the host are among the first lines of traditional antimicrobial defense. Previous work shows no link between a *Wolbachia* infection and the induction of AMPs. Here we compare the expression of protein in a cell line naturally infected with *Wolbachia* and an identical cell line cured of the infection through the use of antibiotics. Protein extracts of each cell line were analyzed by two dimensional gel electrophoresis and LC/MS/MS. Our results show the upregulation of host antioxidant proteins, which are active against ROS generated by aerobic cell metabolism and during an immune response. Furthermore, flow cytometric and microscopic analysis demonstrates that ROS production is significantly greater in *Wolbachia*-infected mosquito cells and is associated with endosymbiont-containing vacuoles located in the host cell cytoplasm. This is the first empirical data supporting an association between *Wolbachia* and the insect antioxidant system.

## Introduction

*Wolbachia* are maternally inherited obligate intracellular gram negative α-proteobacteria closely related to the *Rickettsia.* They were first described in the ovaries of *Culex pipiens*, and are common in insects and filarial nematodes. *Wolbachia* can induce diverse reproductive phenotypes in hosts, including feminization, male-killing, parthenogenesis, and cytoplasmic incompatibility [1]–[4] all of which contribute to the success of infected females at the expense of infected males.

How *Wolbachia* avoid destruction by the host innate immune response is unknown. In *Drosophila,* gram negative bacteria activate the *IMD* pathway inducing the synthesis of potent antimicrobial peptides (AMPs) such as *attacin*, *cecropin*, *drosocin*, and *diptericin* [5]. However, endosymbionts including *Wolbachia* [6] and *Spiroplasma* [7] fail to induce AMP synthesis in their insect hosts, nor do they suppress ectopic immune activation.

The generation of reactive oxygen species (ROS) is among the first lines of defense against invading microbes [8], [9]. ROS, including superoxide radicals, hydrogen peroxide, and hydroxyl radicals are formed as by-products of aerobic metabolism. In vertebrates, following phagocytosis of bacteria, superoxide is produced by an NADPH oxidase complex that assembles at the phagosomal membrane in a reaction called an oxidative burst [10]. From superoxide additional ROS are formed, all of which are active against bacteria [11]. In insects, superoxide generative reactions mimic the oxidative burst seen in vertebrates [12], [13]. In *Anopheles gambiae* high ROS levels generated after a blood meal confer resistance to *Plasmodium* infection [14] and bacterial challenge.

High concentrations of ROS create a state of oxidative stress, resulting in damage to lipids, nucleic acids, and proteins and reducing life span [15]. An unbalanced production of ROS has been implicated in human disease, including atherosclerosis, neurodegenerative and ophthalmologic diseases, and cancer [16]. Complex antioxidant defense systems have evolved to combat damaging ROS [17]. Detoxification of ROS is required for maintaining fecundity in mosquitoes [18], *Drosophila* [19], and mammals [20]. Herbivorous insects have developed defenses against prooxidant allelochemicals from host plants [21].

In order to elucidate mechanisms of host-microbe symbiosis, we have compared protein expression in an *Aedes albopictus* embryonic cell line (Aa23) naturally infected with *Wolbachia* and a parallel cell line cured of *Wolbachia*, using two dimensional polyacrylamide gel electrophoresis (2-D PAGE). Our results show that expression of host antioxidant proteins is induced in Aa23 cells infected with *Wolbachia*. Two bacterial antioxidant proteins were also identified. Futhermore, ROS production is significantly increased in infected cells compared to cured cells. These results illustrate, for the first time, an association between the insect antioxidant pathway and *Wolbachia* infection.

## Results

### PCR analysis of cell lines

**Figure 1:**
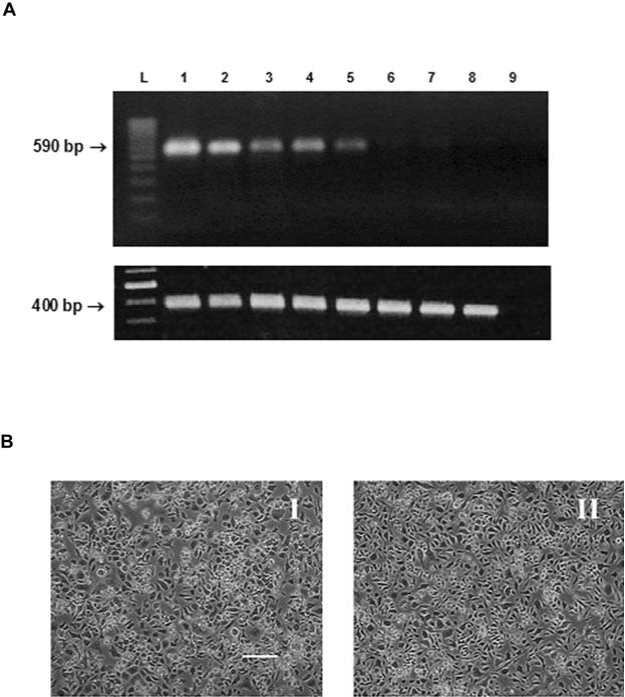
*Wolbachia* stably infects Aa23 cells and can be cured by antibiotic treatment. (A) PCR analysis using *Wolbachia wsp* primers (top) and arthropod 28S primers (bottom) of Aa23 cells treated with 10 ug/ml rifampicin for seven passages. Lane L: molecular ladder. Lane 1: stably infected Aa23 cells. Lanes 2 through 8: cells treated with rifampicin for 1, 2, 3, 4, 5, 6, and 7 passages. Lane 9: negative control. (B) Aa23 cells stably infected with *Wolbachia* (I) and Aa23 cells cured of *Wolbachia* using rifampicin (II). Bar, 100 µm.

DNA from *Wolbachia*-infected Aa23 cells amplified with primers for *Wolbachia* surface protein (*wsp*) generated a band at 590 bp (Figure 1A, top). Rifampicin treatment resulted in the total loss of the *Wolbachia* signal over the course of 7 passages. All DNA extracts produced a band at approximately 400 bp using 28S arthropod primers, confirming DNA template quality (Figure 1A, bottom). The two cell lines are morphologically indistinguishable (Figure 1B).

### Protein induction

**Figure 2:**
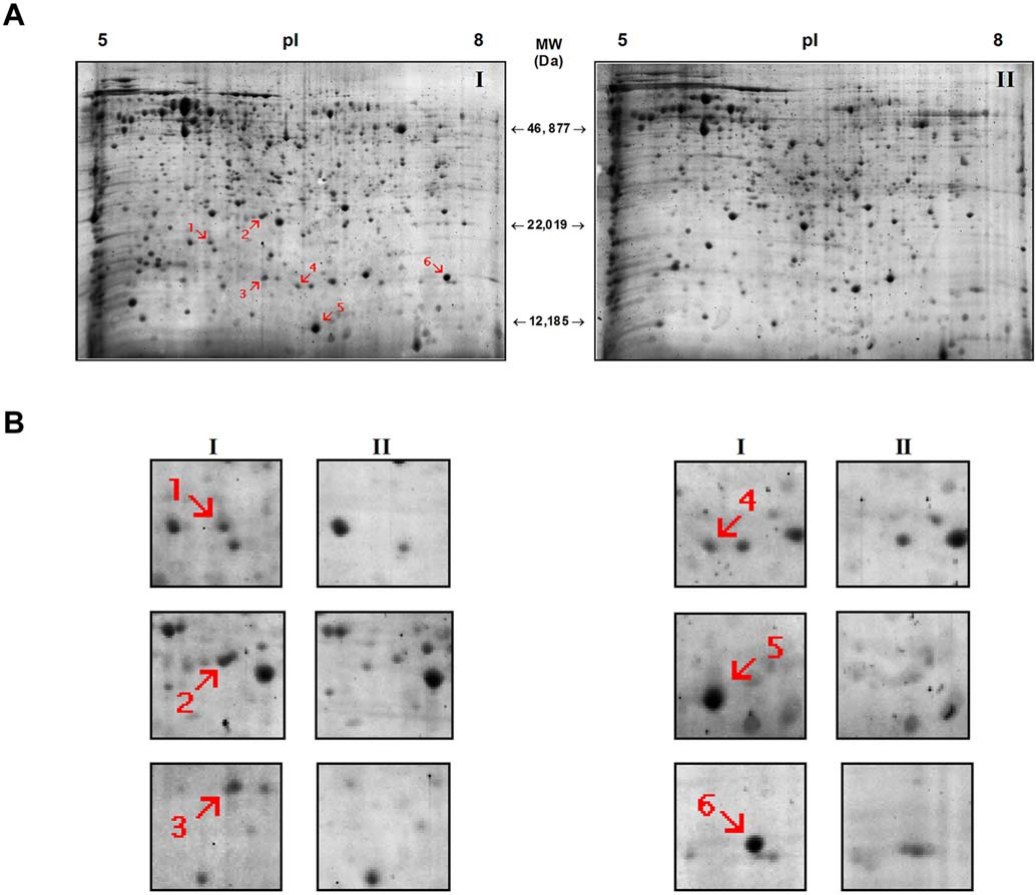
2-D Page of *Wolbachia*-infected and uninfected Aa23 cells. (A) Identification of proteins unique to *Wolbachia-*infected Aa23 cells. Approximately 750 ug of protein extract from an Aa23 cell line stably infected with *Wolbachia* (I) and a parallel cell line cured of a *Wolbachia* infection (II) were analyzed. Proteins expressed only in the presence of a *Wolbachia* infection (ID #1–6) are identified. (B) Gel sections showing proteins selected for LC/MS/MS analysis.

A consistent 2 dimensional profile (Figure 2A) was obtained from protein extracts representing 3 biological replicates (using independently cured Aa23T cell lines). Six proteins (Protein ID #1–6) shown in Figure 2B from *Wolbachia*-infected Aa23 cells failed to appear on the gel from *Wolbachia* – free Aa23 cells. These proteins are antioxidant proteins (Table 1). Proteins 1 (glutathione peroxidase; GPx); 3, 4, 5 (CuZn superoxide dismutase; CuZnSOD) and 6 (peroxiredoxin; Prx) are host proteins. In addition to these, spot 5 contains *Wolbachia* chaperone protein GroES, and spot 3 contains *Wolbachia* bacterioferritin (Bfr). A third *Wolbachia* protein, iron superoxide dismutase (FeSOD) was identified in spot 2.

**Table 1:** Identification of proteins visualized by 2D PAGE.

2D PAGE - Host Protein Matches (*Aedes albopictus*)								
Protein ID	Organism Database	Mowse Score	PI	Mass (Da)	Coverage (%)	Protein	Accession #	Matched Peptides
1	*Aedes aegypti*	99	6.13	19,150	14	Glutathione peroxidase	gi|108871565	K.GNYAELTELSQK.Y R.VNVNGDDAAPLYK.Y
3 *	*Aedes aegypti*	59	5.77	15,616	13	Cu2+/Zn2+ superoxide dismutase	gi|94468490	R.TVVVHADPDDLGLGGHELSK.S
4	*Aedes aegypti*	157	5.77	15,616	32	Cu2+/Zn2+ superoxide dismutase	gi|94468490	K.AVCVLSGDVK.G
								K.VDISDSQISLSGPLSILGR.T
								R.TVVVHADPDDLGLGGHELSK.S
5 *	*Aedes aegypti*	65	5.77	15, 616	12	Cu2+/Zn2+ superoxide dismutase	gi|94468490	K.VDISDSQISLSGPLSILGR.T
6	*Aedes aegypti*	355	6.71	16,862	56	Peroxiredoxin-like protein	gi|55233150	K.VNMADLCAGK.K+Oxidation (M)
								R.YSMVLEDGVIK.S
								R.YSMVLEDGVIK.S+Oxidation (M)
								K.IPSIDLFEDSPANK.V
								K.QLELGADLPPLGGLR.S
								K.VVLFAVPGAFTPGCSK.T
								K.SLNVEPDGTGLSCSLADK.I
								K.EGDKIPSIDLFEDSPANK.V

2D PAGE - Endosymbiont Protein Matches (*Wolbachia pipientis*)								
Protein ID	Organism Database	Mowse Score	PI	Mass (Da)	Coverage (%)	Protein	Accession #	Matched Peptides
2	*Wolbachia (D. mel)*	54	5.98	24,048	10	Fe superoxide dismutase	gi|42520581	M.SFTLPELPYDK.T
								K.IQDDIGGFDK.F
3 *	*Wolbachia (D. mel)*	109	5.74	18,573	22	Bacterioferritin	gi|42521044	K.NELNEELEHANK.L
								K.GVPNFQDTNEISK.H
5 *	*Wolbachia*	314	6.49	8457	79	GroES	gi|21742794	K.TGDKVFYR.Q
								K.ESDLLAVIK.-
								K.GEVIAIGGGSR.N
								K.QGGIVLPSSAEK.K
								K.KPTKGEVIAIGGGSR.N
								R.QWAGTEVEHDNEK.Y
								K.YVVMKESDLLAVIK.-
								K.YVVMKESDLLAVIK.-+Oxidation (M)
								R.QWAGTEVEHDNEKYVVMK.E

Proteins 1–6 are expressed only in the presence of a *Wolbachia* infection. Proteins denoted with a ^*^ match both host and endosymbiont proteins. Protein matches to mosquito (*Ae. aegypti*) and *Wolbachia* are reported, along with the corresponding mowse score, isoelectric point, molecular mass, sequence coverage, protein name, accession number, and predicted matching peptide sequences.

Several antioxidant enzymes exist as multiple isoforms, exhibiting small variations in isolectric point and molecular mass, which are likely the result of post-translational modifications [22], [23]. CuZn SOD is known to undergo phosphorylation [24] and glycosylation [25] in addition to copper and zinc binding [26]. Similarly, phosphorylation of peroxiredoxin [27], and glutathione peroxidase [28] is common. Such modifications would explain the appearance of CuZn superoxide dismutase at three locations (spots 3,4, and 5) within close proximity of one another (Figure 2A).

The presence of the *Wolbachia* chaperonin GroES is not surprising. Expression of this protein in conjunction with its cochaperonin GroEL is common in endosymbiotic bacteria, and is believed to play an essential role in successfully maintaining an intracellular lifestyle by managing deleterious mutations[29].

### Flow cytometric analysis

**Figure 3:**
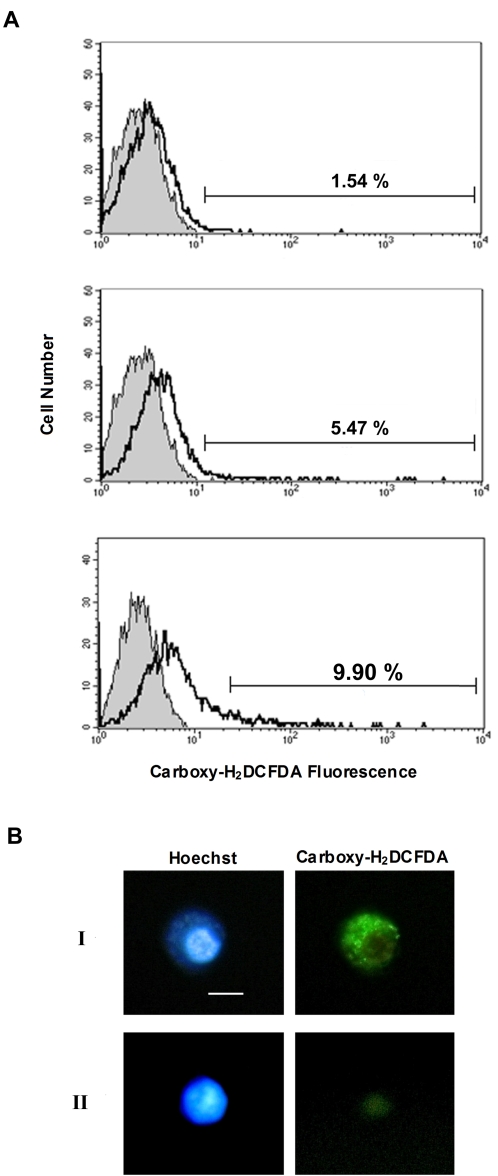
Analysis of ROS formation in *Wolbachia* -infected and uninfected Aa23 cells. (A) Flow cytometric analysis of *Wolbachia* –infected and uninfected Aa23 cells using the fluorescent ROS marker carboxy-H_2_DCFDA. Histograms representative of three replicates are shown. The negative control (shaded) consists of unlabeled cells. Test samples (black lines) include: uninfected Aa23 cells (top panel), uninfected Aa23 cells induced to produce ROS using TBHP (middle panel), and infected Aa23 cells (bottom panel). Carboxy-H_2_DCFDA positive cells are represented on each histogram. (B) Microscopic analysis of *Wolbachia*-infected (I) and uninfected (II) Aa23 cells. Hoechst stain was used to label DNA (left panel). Carboxy-H_2_DCFDA was used to label ROS (right panel). Bar, 10 µm.

The increase in host CuZnSOD, Prx, and GPx levels in response to a *Wolbachia* symbiosis suggests an increase in ROS within this system. To investigate this further we labeled infected and uninfected cells with the fluorescent ROS indicator carboxy-H_2_DCFDA, and evaluated ROS formation by flow cytometry and microscopy. Only 1.54% of uninfected Aa23 cells exhibited ROS formation when examined by flow cytometry (Figure 3A, top). This number rose to 5.47% following induction with TBHP (Figure 3A, middle). In contrast, 9.90% of cells infected with *Wolbachia* fluoresced, demonstrating a substantial increase in ROS formation (Figure 3A, bottom). Microscopic analysis shows that ROS generation is associated with *Wolbachia* in the cytoplasm of Aa23 cells (Figure 3B).

## Discussion

### Host antioxidants

Superoxide dismutases (SOD) are conserved metalloenzymes which catalyze the dismutation of superoxide radicals into hydrogen peroxide and oxygen and are essential in combating oxidative stress [30]. Insects have 3 families of SOD enzymes – a mitochondrial MnSOD and two CuZnSODs, one of which is primarily cytoplasmic and one which is extracellular [31]. The mosquito CuZnSOD identified here (gi|94468490) is homologous to the cytoplasmic *Drosophila* CuZnSOD (gi|17136496) (http://www.ncbi.nlm.nih.gov/blast). Insects deficient in cytoplasmic CuZnSOD suffer from a number of detrimental effects, including a reduction in lifespan and fertility, and an increase in spontaneous DNA damage [32], while insects overexpressing CuZnSOD in combination with catalase show a significant extension in lifespan, emphasizing the physiological importance of these enzymes [33]. Cytoplasmic SOD in the scallop *Chlamys farreri* has been shown to be inducible upon challenge with *Listeria anguillarium* and *Micrococcus luteus* [34].

Peroxiredoxins (Prx) are ubiquitous antioxidant enzymes which reduce peroxides in a thiol dependent manner [35]. Two subgroups have been identified, the 1-Cys and 2-Cys Prxs, based on the number of conserved cysteine residues present [36]. Most 2-Cys Prxs utilize reduced thioredoxin (Trx) as an electron donor and are therefore referred to as thioredoxin peroxidases (TPx) [37]. Prx mutant yeast strains demonstrate a growth rate significantly less than wild-type strains under aerobic conditions, suggesting this group of proteins is essential for cell growth and division [38]. The mosquito Prx induced by *Wolbachia* (gi|55233150) in this study shares 58% sequence identity with human Prx-5 (gi|83405871) (http://www.ncbi.nlm.nih.gov/BLAST) and further investigation is required to characterize its antioxidant function.

Glutathione peroxidase (GPx) (gi|108871563) expression is also enhanced in *Wolbachia* infected cells. GPx catalyzes the reduction of hydrogen peroxide and organic hydroperoxides using reduced glutathione (GSH) as an electron donor [39]. The flavoenzyme glutathione reductase (GR) recycles the oxidized form of glutathione, glutathione disulfide (GSSH) back to its reduced form, maintaining high concentrations of GSH [40]. *Drosophila melanogaster* lacks a functional GR which seems to support early findings that insects lack GPx activity [41], [42]. However, Trx is capable of reducing GSSH, suggesting that a Trx system compensates for the absence of GR in *Drosophila* [42]. The *Drosophila* genome contains two GPx homologs [43], one of which utilizes reduced Trx as an electron donor; as a result, it has been described as a GPx homolog with TPx (thioredoxin peroxidase) activity (GTPx-1) [44]. GTPx-1 maintains a key role in the oxidative stress response [44]. The second GPx homolog of *Drosophila* (termed-GPx-like) has not yet been biochemically characterized [44].

### Endosymbiont antioxidants

Intracellular free iron reacts with H_2_O_2_ to generate highly reactive hydroxyl radicals. Superoxide radicals can destroy enzymes containing Fe-S clusters, and release additional free iron into the intercellular environment [45]. Ferritins are a broad superfamily of iron-storage proteins common to both aerobic and anaerobic organisms and are essential components of antioxidant pathways [46]. Iron is sequestered by ferritins and strictly regulated under normal circumstances and during times of oxidative stress. Although ferritins generally lack haem groups, some bacterial forms are haem-containing, and are referred to as bacterioferritins (Bfr) [47]. Like their eukaryotic homologs, Bfr are integral factors in bacterial iron storage and in combating iron-mediated oxidative stress [48]. Bfr mutants of *P. aeruginosa* demonstrate an increased sensitivity to peroxides, emphasizing the importance of Bfr in the redox stress response [49]. Our data suggest that *Wolbachia* Bfr (gi|42521044) may protect the endosymbiont from ROS generated in its intracellular compartment.

Bacterial superoxide dismutase (Fe-SOD) contains iron at its catalytic center and plays an important role in the pathogenesis of numerous bacteria, including *Shigella flexneri* [50], *Pseudomonas aeruginosa* [51] and *Bordetella pertussis* [52]. In *E. coli*, Fe-SOD is constitutively expressed, while two other forms, a manganese-containing form (MnSOD) and a copper and zinc-containing form (CuZnSOD) are induced in response to oxygen [53], [54] . In contrast to *E. coli*, the genome of the *Wolbachia* endosymbiont of *Drosophila melanogaster* (*w*Mel) has only one SOD gene, which corresponds to the FeSOD identified here (gi|42520581). This enzyme likely plays a fundamental role in the management of oxidative stress within the vacuoles harboring *Wolbachia*.

### Antioxidant pathways and the endosymbiont

For the first time, we report a significant increase in ROS which may be a host-mediated immune response to *Wolbachia* or, alternatively, may be generated by the aerobic metabolism of *Wolbachia* themselves [55]. In either case, increased host antioxidant expression is an adaptation to symbiosis and our evidence suggests that the neutralization of potentially deadly ROS by elevated antioxidant levels is important for maintaining this unique relationship.

The *E. coli* genome has numerous antioxidant proteins, including three SOD's – a CuZnSOD [56], an MnSOD, and an FeSOD [57], two catalases [58], four peroxidases – GPx [59], thiol peroxidase [60], bacterioferritin comigratory protein [61], alkyl hydroperoxide reductase [62], and Bfr [63] (http://wishart.biology.ualberta.ca/BacMap/) [64]. The genome of *w*Mel contains four homologs of these ten *E. coli* proteins - FeSOD, Bfr, bacterioferritin comigratory protein, and alkyl hydroperoxide reductase (http://www.ncbi.nlm.nih.gov/blast). *Wolbachia*, like most obligate endosymbionts, has a reduced genome [55], the result of an ongoing adaptation to an intracellular lifestyle. The induction of host antioxidant proteins by *Wolbachia* may be an adaptive mechanism for coping with ROS despite having lost genes coding for bacterial antioxidants.

*R. rickettsii*, the agent of Rocky Mountain Spotted Fever, stimulates the production of ROS in mammalian endothelial cells and inflicts host cell damage via lipid peroxidation of membranes [65]. Expression of host antioxidants is modified in a manner that is consistent with the generation of intracellular peroxides [66]–[68]. Such evidence lends support to the premise that *Wolbachia* manipulates host antioxidant systems in a manner that is beneficial to its survival.

Microarray analyses using *Drosophila* have found little induction of host antioxidant transcription in response to pathogenic bacteria (*E. coli* and *M. luteus*) or fungi (*B. bassiana*). While one study [69] discovered a single transcript induced in response to septic injury (a peroxidase), a second study identified none [70]. Recently, DNA microarray analysis of *Drosophila* S2 cells infected with *Wolbachia* demonstrated an increase in transcripts belonging to the *Toll* and *Imd* immune signaling pathways, and the antimicrobial peptides *attacin* and *diptericin* [71]. In this experiment the *Wolbachia* were introduced artificially using the shell vial technique and the infection was subsequently lost after 18 passages. In contrast, our results show that a stable symbiotic interaction with *Wolbachia* in mosquito cells involves ROS generation and induction of antioxidant enzymes. Although the role of ROS and antioxidants in maintaining symbiosis *in vivo* has not yet been determined, it will be interesting to see if these pathways differentiate between insects that can establish a long term association with symbionts and those that cannot.

## Materials and Methods

### Cell culture

The Aa23 cell line was provided by Steven Dobson and cultured as previously described [72]. The growth medium consisted of equal volumes of Mitsuhashi-Maramorosch (MM) and Schneider's Insect medium (Sigma) supplemented with 20% heat-inactivated fetal bovine serum (Sigma). Three uninfected cell lines were independently generated from the original cell line by adding 10 µg/ml rifampicin to the culture medium for 7 passages [73].

### PCR analysis

PCR analysis using *Wolbachia wsp* primers [74] confirmed the presence or absence of *Wolbachia*. DNA was isolated using the Sigma GenElute Mammalian Genomic DNA MiniPrep kit and stored until use at −20°C. PCR conditions have been previously described [75]. The presence of *Wolbachia* was confirmed by the existence of a 590 bp product. Universal 28S ribosomal DNA primers D3A and D3B [76] produced a band at 400 bp, confirming DNA template quality.

### Protein purification

Protein extracts were prepared as described by Adrain *et al.* [77]. From each cell line, 5×10^8^ cells were packed into a 2-ml Dounce homogenizer with an equal volume of ice-cold cell extract buffer (CEB: 20 mM HEPES-KOH, pH 7.5, 1.5 mM MgCl_2_, 1 mM EDTA, 1 mM EGTA, 1 mM DTT, 250 mM sucrose, 10 mM KCl, 100 µM phenylmethylsulfonyl fluoride, 10 µg/ml leupeptin, 2 µg/ml aprotinin). Cells were incubated for 20 minutes on ice then homogenized. The homogenate was centrifuged for 15 minutes at 15,000× g. The pellet was resuspended in ice-cold CEB with 6% IGEPAL CA-630 (Sigma), and again subjected to the extraction procedure previously described. The supernatant was collected, and a Bradford Assay was used to determine the protein concentration (µg/µl). Protein (∼750 µg) was precipitated in acetone overnight, and cleared of contaminants using the Bio-Rad 2-D Clean-Up Kit.

### 2 dimensional PAGE

Protein pellets were suspended in 322 µl of Amersham DeStreak Rehydration Solution containing 0.5% Amersham IPG buffer (pH 3–10 NL) and 10 mM DTT, then loaded onto 17 cm (pH 5–8) Bio-Rad ReadyStrip IPG strips and rehydrated passively overnight at room temperature. Isoelectric focusing was performed on an Amersham IPGphor Isoelectric focusing unit. Following equilibration for 15 minutes in each of two equilibration buffers (50 mM Tris-HCl, pH 8.8, 6M urea, 30% glycerol, 2% SDS, 0.002% bromophenol blue), one containing 1% DTT, the other containing 2.5% IAA, the strips were run on 12% homogenous SDS-PAGE gels with a 4% stacking gel at 280V for 4 hours. Gels were stained with Deep Purple (Amersham) and imaged on a Fugifilm FLA-500 scanner at 473 nm, then stained in Coomassie Blue (Sigma).

### Protein identification

Protein spots were excised manually and analyzed by LC/MS/MS at the Southern Alberta Mass Spectrometry (SAMS) Centre for Proteomics at the University of Calgary. Matching protein sequences were identified by the MASCOT search engine (www.matrixscience.com). From the significant hits generated by MASCOT, those corresponding to the primary sequence databases of *Ae. Aegypti* and *w*Mel were considered most probable.

### ROS flow cytometric analysis

The presence of ROS within *Wolbachia*-infected and uninfected Aa23 cells was evaluated using the fluorogenic marker carboxy-H_2_DCFDA (Molecular Probes). Prior to labeling, cells were suspended in PBS containing 1.26 mM CaCl_2_, 0.81 mM MgSO_4_ and 5 mM EDTA (Buffer A). For measurement of ROS, cells were incubated in Buffer A containing 25 µM of carboxy-H_2_DCFDA for 30 min at 27°C, washed twice in Buffer A, and analyzed immediately. Positive controls were generated by incubating cells in 150 µM *tert*-butyl hydroperoxide (TBHP) in cell culture medium for 90 min followed by labeling. A total of 10 000 events were aquired using the CellQuest software on a FACScan flow cytometer (Beckton Dickinson).

### Microscopic ROS assay

Infected and uninfected Aa23 cells were permitted to adhere to glass slides, labeled as described above using carboxy-H_2_DCFDA, and counterstained with Hoechst 333342. Slides were imaged on a Zeiss Axiomat 40 fluorescent microscope with a Canon PowerShot camera.
